# Supplementary material for: The effects of mismatched train and test data cleaning pipelines on regression models: lessons for practice
Source: PeerJ Comput Sci. 2025 Apr 2;11:e2793. doi: 10.7717/peerj-cs.2793 (PMC12190241; doi:10.7717/peerj-cs.2793)
Supplement: Supplemental Information 1 [file peerj-cs-11-2793-s001.zip › mismatched_data_cleaning-main/analysis/appendix.pdf]

Appendix of 'The effects of mismatched train and test data cleaning pipelines on regression models: lessons for practice'

Cleaning Setups - Airbnb Data

Setup number	mv_repair	outlier_detection	outlier_repair	duplicate_repair
0	delete	none	NA	NA
1	delete	none	NA	key_val
2	delete	SD	mean	NA
3	delete	SD	mean	key_val
4	delete	SD	median	NA
5	delete	SD	median	key_val
6	delete	SD	mode	NA
7	delete	SD	mode	key_val
8	delete	IQR	mean	NA
9	delete	IQR	mean	key_val
10	delete	IQR	median	NA
11	delete	IQR	median	key_val
12	delete	IQR	mode	NA
13	delete	IQR	mode	key_val
14	mean-mode	none	NA	NA
15	mean-mode	none	NA	key_val
16	mean-mode	SD	mean	NA
17	mean-mode	SD	mean	key_val
18	mean-mode	SD	median	NA
19	mean-mode	SD	median	key_val
20	mean-mode	SD	mode	NA
21	mean-mode	SD	mode	key_val
22	mean-mode	IQR	mean	NA
23	mean-mode	IQR	mean	key_val
24	mean-mode	IQR	median	NA
25	mean-mode	IQR	median	key_val
26	mean-mode	IQR	mode	NA
27	mean-mode	IQR	mode	key_val
28	median-mode	none	NA	NA
29	median-mode	none	NA	key_val
30	median-mode	SD	mean	NA
31	median-mode	SD	mean	key_val
32	median-mode	SD	median	NA
33	median-mode	SD	median	key_val
34	median-mode	SD	mode	NA
35	median-mode	SD	mode	key_val

36	median-mode	IQR	mean	NA
37	median-mode	IQR	mean	key_val
38	median-mode	IQR	median	NA
39	median-mode	IQR	median	key_val
40	median-mode	IQR	mode	NA
41	median-mode	IQR	mode	key_val
42	mode-mode	none	NA	NA
43	mode-mode	none	NA	key_val
44	mode-mode	SD	mean	NA
45	mode-mode	SD	mean	key_val
46	mode-mode	SD	median	NA
47	mode-mode	SD	median	key_val
48	mode-mode	SD	mode	NA
49	mode-mode	SD	mode	key_val
50	mode-mode	IQR	mean	NA
51	mode-mode	IQR	mean	key_val
52	mode-mode	IQR	median	NA
53	mode-mode	IQR	median	key_val
54	mode-mode	IQR	mode	NA
55	mode-mode	IQR	mode	key_val
56	mean-dummy	none	NA	NA
57	mean-dummy	none	NA	key_val
58	mean-dummy	SD	mean	NA
59	mean-dummy	SD	mean	key_val
60	mean-dummy	SD	median	NA
61	mean-dummy	SD	median	key_val
62	mean-dummy	SD	mode	NA
63	mean-dummy	SD	mode	key_val
64	mean-dummy	IQR	mean	NA
65	mean-dummy	IQR	mean	key_val
66	mean-dummy	IQR	median	NA
67	mean-dummy	IQR	median	key_val
68	mean-dummy	IQR	mode	NA
69	mean-dummy	IQR	mode	key_val
70	median-dummy	none	NA	NA
71	median-dummy	none	NA	key_val
72	median-dummy	SD	mean	NA
73	median-dummy	SD	mean	key_val
74	median-dummy	SD	median	NA
75	median-dummy	SD	median	key_val
76	median-dummy	SD	mode	NA
77	median-dummy	SD	mode	key_val
78	median-dummy	IQR	mean	NA
79	median-dummy	IQR	mean	key_val
80	median-dummy	IQR	median	NA
81	median-dummy	IQR	median	key_val

82	median-dummy	IQR	mode	NA
83	median-dummy	IQR	mode	key_val
84	mode-dummy	none	NA	NA
85	mode-dummy	none	NA	key_val
86	mode-dummy	SD	mean	NA
87	mode-dummy	SD	mean	key_val
88	mode-dummy	SD	median	NA
89	mode-dummy	SD	median	key_val
90	mode-dummy	SD	mode	NA
91	mode-dummy	SD	mode	key_val
92	mode-dummy	IQR	mean	NA
93	mode-dummy	IQR	mean	key_val
94	mode-dummy	IQR	median	NA
95	mode-dummy	IQR	median	key_val
96	mode-dummy	IQR	mode	NA
97	mode-dummy	IQR	mode	key_val

Table 1: Numbering of cleaning pipelines for Airbnb dataset.
